# MexXY-OprM efflux pump mediates resistance to odilorhabdins in *Pseudomonas aeruginosa*

**DOI:** 10.1128/spectrum.00466-26

**Published:** 2026-06-16

**Authors:** Emilie Racine, Lucile Pantel, Kelly Maurent, Hazel L. S. Fuchs, Matthieu Sarciaux, Jessica Houard, Bettina Hinkelmann, Marie Attwood, Cédric Muller, Sophie Guénard, Alan Noel, Alasdair MacGowan, Alain Givaudan, Mark Brönstrup, Maxime Gualtieri

**Affiliations:** 1 Nosopharm, Nîmes, France; 2 Department of Chemical Biology, Helmholtz Centre for Infection Research 28336, Braunschweig, Germany; 3 Bristol Centre for Antimicrobial Research and Evaluation (BCARE), Infection Sciences, Southmead Hospital, Bristol, United Kingdom; 4 Smaltis, Bioinnovation, Rue Charles Bried, Besançon, France; 5 DGIMI, Univ Montpellier, INRAE, Montpellier, France; 6 German Center for Infection Research (DZIF), Site Hannover-Braunschweig, Braunschweig, Germany; 7 Center for Biomolecular Drug Research (BMWZ), Hannover, Germany; 8 UPR CHROME - Nîmes University, Nîmes, France; Oklahoma State University, Stillwater, Oklahoma, USA

**Keywords:** odilorhabdins, *Pseudomonas aeruginosa*, MexXY-OprM, efflux pump-mediated resistance, ribosome-targeting antibiotics, antimicrobial resistance

## Abstract

The urgent need for new antibiotic classes to treat infections caused by multidrug-resistant gram-negative pathogens has driven interest in odilorhabdins (ODLs), a novel class of cationic antimicrobial peptides that target the bacterial ribosome. NOSO-502, the first clinical candidate in this family, exhibits potent activity against multidrug-resistant *Enterobacteriaceae* but is inactive against *Pseudomonas aeruginosa* isolates. This study investigates the intrinsic resistance of *P. aeruginosa* to NOSO-502 using the reference strain PAO1. Our data indicate that NOSO-502 induces overexpression of the MexXY-OprM efflux pump, which mediates drug extrusion from the periplasm and limits antibacterial activity. To further explore the role of MexXY-OprM, we evaluated NOSO-95C, the first purified ODL from natural origin. Unlike NOSO-502, NOSO-95C does not induce overexpression of MexXY-OprM at subinhibitory concentrations, resulting in measurable antibacterial activity against PAO1. However, NOSO-95C remains susceptible to MexXY-OprM-mediated efflux, as evidenced by its limited efficacy against strains that constitutively overexpress this pump. To better understand the structural basis of these differences, we investigated the relationship between ODL structures and the ability to induce *mexXY* expression. Our results demonstrate that both the amino acid at position 7 and the *C*-terminal region of the ODL scaffold are critical determinants of MexXY induction and efflux susceptibility. These findings offer valuable insights for the design of next-generation ODLs with improved efficacy against *P. aeruginosa*.

**IMPORTANCE:**

*Pseudomonas aeruginosa* is a major cause of hospital-acquired infections. Odilorhabdins (ODLs) are a novel class of ribosome-targeting antibiotics with promising activity against multidrug-resistant gram-negative bacteria. Unfortunately, their efficacy against *P. aeruginosa* remains limited. In this study, we identify the MexXY-OprM efflux pump as the primary determinant of intrinsic resistance of *P. aeruginosa* to ODLs. We show that the clinical candidate NOSO-502 strongly induces mexXY expression, resulting in active self-efflux and loss of antibacterial activity, whereas the natural ODL NOSO-95C displays reduced induction and partial activity. By combining microbiological, transcriptional, uptake, and structure-activity analyses, we demonstrate that specific structural patterns of ODLs govern both efflux susceptibility and efflux pump induction. These findings pave the way for the design of the next ODL generation with improved activity against *P. aeruginosa*.

## INTRODUCTION

The emergence and spread of bacteria resistant to most or all existing antibiotics is a major public health concern and could lead to the next global pandemic (1, 2). The World Health Organization (WHO) has classified carbapenem-resistant *Pseudomonas aeruginosa* as a high-priority pathogen, highlighting the urgent need for new anti-infective agents (3). *P. aeruginosa* is an opportunistic gram-negative pathogen responsible for a wide range of severe nosocomial infections and is a leading cause of chronic lung infections in cystic fibrosis patients (4, 5). This situation underlines the pressing need for innovation in antibiotic discovery (6, 7).

Odilorhabdins (ODLs) are cationic antimicrobial peptides that represent a novel class of ribosome-targeting antibiotics. They bind to the 30S ribosomal subunit near the decoding center, thereby disrupting translation fidelity (8). The first ODLs, including NOSO-95A, B, and C, were isolated from *Xenorhabdus nematophila* cultures (8). NOSO-95C displays moderate activity against gram-negative pathogens such as *Escherichia coli* and *P. aeruginosa* (8). Medicinal chemistry optimization led to the development of NOSO-502, a compound with potent activity against multidrug-resistant *Enterobacteriaceae* that has completed preclinical development (9, 10). However, NOSO-502 shows limited efficacy against *P. aeruginosa* (11, 12).

Previous studies have linked efflux pump overexpression to reduced ODL susceptibility in several gram-negative species. In *Klebsiella pneumoniae* and *Enterobacter cloacae* complex strains, NOSO-502 resistance has been associated with mutations in the CrrAB two-component system, leading to overexpression of the KexD efflux pump (13, 14). Other mechanisms, such as point mutations in the ribosomal protein S10 and *N*-acetylation of ODLs, have also been implicated in resistance (8, 15).

In this study, we investigate the mechanisms underlying the low susceptibility of *P. aeruginosa* PAO1 to ODLs, including NOSO-502 and NOSO-95C. We demonstrate that these compounds are substrates of the MexXY-OprM efflux pump and that their ability to induce expression of this system correlates with the observed resistance phenotype.

## RESULTS

### Susceptibility of *P. aeruginosa* isolates to ODLs

We evaluated the antibacterial activity of two ODLs, NOSO-502 and NOSO-95C, and comparators against a panel of 15 *P. aeruginosa* strains. This panel was composed of clinical and reference strains, including isolates resistant to imipenem, gentamicin, ciprofloxacin, tigecycline, or cefepime. Our results showed that all tested strains exhibited a high-level resistance to NOSO-502 (minimum inhibitory concentration [MIC₅₀] ≥64 µg/mL, MIC₉₀ ≥64 µg/mL, range of MIC: ≥64 to ≥64 µg/mL). In contrast, susceptibility to NOSO-95C varied greatly depending on the strain (MIC₅₀ = 16 µg/mL, MIC₉₀ ≥64 µg/mL, range of MIC: 4 to ≥64 µg/mL) (Table 1).

**TABLE 1:** MICs of NOSO-502, NOSO-95C, and antibiotics against reference and clinical strains of *P. aeruginosa* from French hospitals^*a*^

*P. aeruginosa* strains	MIC (µg/mL)						
	NOSO-502	NOSO-95C	GEN	CFP	IPM	CIP	TIG
ATCC27853	≥64	4	1	2	1	0.125	8
CIP 106816	≥64	16	2	8	1	0.125	32
CIP 106817	≥64	32	≥64	≥64	≥64	≥64	≥64
CIP 106880	≥64	≥64	≥64	≥64	32	32	≥64
NCTC 13437	≥64	16	2	8	2	0.5	≥64
1003 682 729	≥64	8	1	2	2	0.125	32
1004 049 478	≥64	4	1	2	8	16	32
1004 091 973	≥64	8	1	2	4	4	32
1004 131 924	≥64	4	0.5	1	0.25	0.125	2
1004 141 296	≥64	8	2	4	0.5	0.125	2
1004 75 25	≥64	16	2	4	2	32	32
2004 149 316	≥64	8	1	8	2	1	≥64
35170	≥64	≥64	≥64	≥64	32	16	≥64
401681	≥64	≥64	32	16	2	1	≥64
5618	≥64	16	4	8	≥64	16	≥64
MIC_50_ (µg/mL)	≥64	16	2	8	2	1	32
MIC_90_ (µg/mL)	≥64	≥64	≥64	≥64	≥64	32	≥64
Range (µg/mL)	≥64 to ≥64	4 to ≥64	1 to ≥64	1 to ≥64	0.25 to ≥64	0.125 to ≥64	2 to ≥64

^*a*^ List of antibiotics: gentamicin (GEN), cefepime (CFP), imipenem (IPM), ciprofloxacin (CIP), and tigecycline (TIG).

To further investigate these differences in susceptibility, the PAO1 strain was chosen as a model. Two variants of this strain were tested: PAO1 (DSM 22644) and PAO1-Bes (Besancon laboratory strain). Notably, the MIC of NOSO-502 against PAO1 was ≥512 µg/mL, whereas the MIC of NOSO-95C was 8 µg/mL. Similar results were obtained with PAO1-Bes, for which the MICs of NOSO-502 and NOSO-95C were ≥512 µg/mL and 4 µg/mL, respectively (Table 2). Although genetically closely related, laboratory PAO1 strains can exhibit phenotypic differences due to genetic drift, spontaneous mutations, or repeated subculturing (16). By testing both strains, we aimed to ensure that the observed activity of ODLs was not restricted to a single PAO1 isolate but representative of the broader PAO1 genetic background.

**TABLE 2:** Antibacterial activity of NOSO-502, NOSO-95C, gentamicin (GEN), and ciprofloxacin (CIP) against *P. aeruginosa* strains including wild-type strain PAO1, PAO1 efflux-deficient mutants (PAO750, 11B, PAO1T, and PAO1ΔmexXY), PAO1 mutants overexpressing efflux pump systems (4098E, ERYR, PAO7H, and PAOW2), and PAO1 with mutation in regulatory pathways of *mexXY* expression (PAO1ΔparRS, PAO1ΔarmZ, PAO1 ΔPA5470, and PAO1ΔmexZ)

Strain	Relevant characteristics	MIC (µg/mL)			
		NOSO-502	NOSO-95C	GEN	CIP
PAO1	Wild-type reference strain DSM22644	≥512	8	2	0.125
PAO1Bes	Wild-type reference strain from Besançon laboratory (17)	≥512	4	1	0.125
PA0750	PAO1 efflux-defective, ΔmexAB-oprM, ΔmexCD-oprJ, ΔmexEF-oprN, ΔmexJK, ΔmexXY, and ΔopmH (18)	2	2	0.125	<0.03
11B	PAO1 MexXY-OprM efflux-defective mexX::Tn501 insertion (19)	2	2	0.125	0.125
PAO1T	PAO1 MexXY-OprM efflux-defective oprM::ΩHgr interposon (20)	2	2	0.125	<0.03
4098E	PAO1 overproducing MexAB-OprM nalB mutation (21)	≥512	8	1	0.5
ERYR	PAO1 overproducing MexCD-OprJ nfxB mutation (C199T) (20)	≥512	8	1	0.125
PAO7H	PAO1 overproducing MexEF-OprN nfxC mutation (22)	≥512	8	1	0.5
PAOW2	PAO1Bes overproducing MexXY-OprM ParR mutation (M59I) (17)	≥512	32	4	0.5
PAO1ΔmexXY	PAO1Bes with in-frame deletion of operon mexXY (23)	2	2	0.125	0.125
PAO1ΔparRS	PAO1Bes with in-frame deletion of operon parRS (17)	256	4	2	0.125
PAO1ΔarmZ	PAO1Bes with in-frame deletion of gene PA5471 (17)	≥512	1	0.25	0.125
PAO1 ΔPA5470	PAO1Bes with in-frame deletion of gene PA5470 (17)	≥512	4	1	0.125
PAO1ΔmexZ	PAO1Bes with in-frame deletion of gene mexZ (17)	>512	32	4	0.5

### High intrinsic resistance to NOSO-502 in *P. aeruginosa* PAO1 is mediated by MexXY-OprM

In *P. aeruginosa,* adaptive resistance to antibiotics is frequently associated with the inducible expression of multidrug resistance (MDR) efflux pumps capable of exporting a broad range of compounds (24). Previous observations in *K. pneumoniae* and *Enterobacter cloacae* also suggested that active efflux may limit NOSO-502 activity (13, 14). We therefore investigated whether efflux contributes to intrinsic NOSO-502 resistance in *P. aeruginosa* using efflux-defective PAO1 strains (24).

A >256-fold reduction in NOSO-502 MIC (from ≥512 to 2 µg/mL) was observed in strain PAO750, a PAO1 mutant lacking five major RND-type efflux systems (MexAB-OprM, MexCD-OprJ, MexEF-OprN, MexJK, and MexXY) (Table 2). Among these, MexXY is an inducible efflux system particularly involved in resistance to ribosome-targeting antibiotics, such as NOSO-502 (25). It functions in conjunction with OprM to form a tripartite pump (26). Notably, mutants lacking either *mexXY* or *oprM* (strains 11B and PAO1T) also showed a drastic reduction in MIC (2 µg/mL). The similar MIC values observed in the multi-efflux-deficient mutant (PAO750) and the *mexXY/oprM*-deficient strains support the conclusion that the MexXY-OprM system is the principal efflux mechanism responsible for intrinsic resistance of PAO1 to NOSO-502.

### NOSO-95C exhibits antibacterial activity against *P. aeruginosa* PAO1 but remains susceptible to MexXY-OprM efflux

The role of efflux was then studied using NOSO-95C. As previously observed, NOSO-95C was more active than NOSO-502 against *P. aeruginosa* PAO1 (MIC of 4–8 µg/mL). Nevertheless, *P. aeruginosa* PAO750 and *mexXY*- or *oprM*-defective PAO1 mutants (strains 11B and PAO1T) exhibited increased susceptibility to NOSO-95C (MIC = 2 µg/mL), indicating that efflux by MexXY-OprM also mediates a moderate reduction in susceptibility to NOSO-95C in *P. aeruginosa* PAO1 (Table 2).

The antibacterial activity of NOSO-95C was then evaluated against *P. aeruginosa* PAO1 mutants overproducing MexAB-OprM (strain 4098E), MexCD-OprJ (strain ERYR), MexEF-OprN (strain PAO7H), and MexXY-OprM (strain PAOW2) efflux systems. A decrease in susceptibility to NOSO-95C was only conferred in the mutant overexpressing MexXY-OprM (MIC = 32 µg/mL), showing that NOSO-95C remains a substrate for this efflux pump (Table 2).

### Berberine, a MexXY-OprM inhibitor, increases susceptibility of *P. aeruginosa* PAO1 to NOSO-502 and NOSO-95C

The impact of the efflux pump inhibitors (EPI) berberine (BER) and phenylalanine-arginine-β-naphthylamide (PAβN) was assessed on the antibacterial activity of NOSO-502, NOSO-95C, the aminoglycoside GEN, and the fluoroquinolone CIP against *P. aeruginosa* PAO1 and the *mexXY*-defective PAO1 mutant *P. aeruginosa* 11B. Berberine is a MexXY-OprM inhibitor that attenuates resistance to aminoglycosides (AG) in *P. aeruginosa,* while PAβN has been described as a broad-spectrum EPI in various gram-negative bacteria affecting MexAB-OprM, MexCD-OprJ, and MexEF-OprM, resulting in an increased susceptibility to CIP (27, 28). Addition of 256 µg/mL of berberine reduced the MIC values of NOSO-502 (>4-fold), NOSO-95C (4-fold), and GEN (4-fold) against *P. aeruginosa* PAO1, while no effect was observed against *P. aeruginosa* 11B with these three compounds or with CIP against both strains (Table 3). The addition of 50 µg/mL of PAβN induced a 2-fold to 4-fold increase in NOSO-502, NOSO-95C, and GEN MIC values against *P. aeruginosa* PAO1 and 11B, while the MIC of CIP (used as a control) was decreased 4-fold against both strains (Table 3). As previously observed with AG (29), efflux of ODLs from *P. aeruginosa* is MexXY-OprM-mediated, while PAβN antagonizes the anti-*Pseudomonas* activity of ODLs.

**TABLE 3:** Antibacterial activity of NOSO-502, NOSO-95C, GEN, and CIP against *P. aeruginosa* PAO1 and 11B in the presence of 256 µg/mL of BER, 50 µg/mL of phenylalanine-arginine β-naphthylamide (PAβN), or 128 µg/mL of NOSO-502^*a*^

Strain	Adjuvant	MIC (µg/mL)			
		NOSO-502	NOSO-95C	GEN	CIP
PAO1		>2,048	8	2	0.125
	+ BER	512	2	0.5	0.125
	+ PAβN	>2,048	32	4	0.03
	+ NOSO-502	–	32	8	0.25
11B	–	2	2	0.125	0.125
	+ BER	2	2	0.125	0.125
	+ PAβN	4	4	0.25	0.03

^*a*^ “–” Symbol in the column (Adjuvant) indicate that the experiment was performed without adjuvant, whereas the “–” symbol in the column (NOSO-502) indicate that NOSO-502 was not tested.

### NOSO-502 and NOSO-95C differentially induce the expression of *mexXY* in *P. aeruginosa*

**Fig 1:**
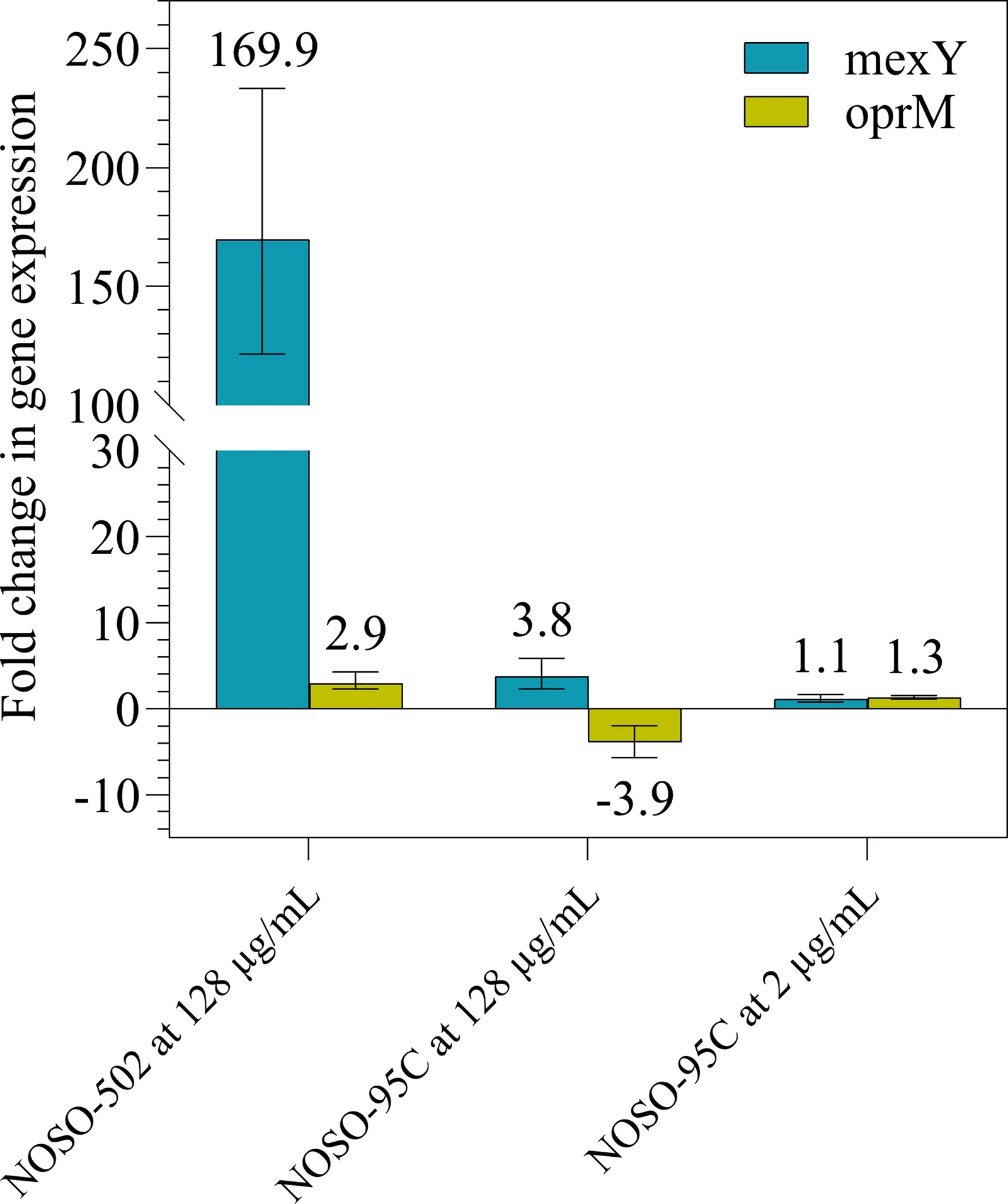
RT-qPCR comparative analysis of *mexY* and *oprM* genes, differentially expressed in PAO1 cultures treated with NOSO-502 or NOSO-95C (128 µg/mL or 2 µg/mL, 45 min) relative to the culture without treatment. Average values of three biological replicates are depicted; the error bars show the SEM.

Expression of the *mexXY* operon is known to be induced by ribosome-targeting antibiotics or ribosomal mutations (25). To assess whether NOSO-502 stimulates this response, we measured the transcript levels of *mexY* (as a proxy for *mexXY*) and *oprM* by RT-qPCR after 45 min of treatment of *P. aeruginosa* PAO1 with 128 µg/mL of NOSO-502 (this concentration corresponds to ≤MIC/4). NOSO-502 induced a strong increase in *mexY* mRNA levels (170-fold) and a moderate increase in *oprM* expression (2.9-fold) compared to untreated cells (Fig. 1).

To determine whether this induction correlates with antibacterial activity, *mexY* and *oprM* expressions were also evaluated in cultures treated with NOSO-95C, either at the same high concentration (128 µg/mL) or at a subinhibitory concentration (2 µg/mL, i.e., MIC/4). NOSO-95C caused only a modest increase in *mexY* expression (3.8-fold) and a decrease in *oprM* levels (3.9-fold) at 128 µg/mL, while no significant changes were observed at 2 µg/mL. These results suggest that the ability to induce *mexXY-oprM* expression differs markedly between NOSO-502 and NOSO-95C, which may contribute to their distinct MIC values against *P. aeruginosa* PAO1.

To assess whether NOSO-502-treated cells overproduce a functional MexXY-OprM efflux system, we measured the MICs of gentamicin (GEN), a known MexXY-OprM substrate, and NOSO-95C in the presence or absence of 128 µg/mL NOSO-502. Consistent with efflux pump induction, we observed a 4-fold increase in the MICs of both GEN and NOSO-95C upon NOSO-502 treatment (Table 3), confirming an enhanced MexXY-OprM activity under these conditions.

### Contribution of ArmZ, MexZ, and ParRS to the intrinsic resistance of *P. aeruginosa* to odilorhabdins

Several regulatory pathways leading to *mexXY* upregulation have been described in *P. aeruginosa*, resulting in 2-fold to 16-fold increases in resistance to MexXY-OprM substrates such as aminoglycosides, cefepime, and fluoroquinolones (17, 30). These pathways include: (i) mutations in *mexZ*, a gene encoding the repressor of the *mexXY* operon; (ii) induction of *armZ*, which encodes an anti-repressor that neutralizes MexZ, typically in response to ribosome-disrupting antibiotics; (iii) activation of the AmgRS two-component system (TCS), either due to genetic mutations or upon exposure to aminoglycosides, which can indirectly promote *mexXY* expression; and (iv) amino acid substitutions in the ParRS TCS, also linked to increased *mexXY* expression (17, 25, 31–34).

To dissect the respective contributions of MexZ, ArmZ, and ParRS to the intrinsic resistance of *P. aeruginosa* to odilorhabdins, we tested single-deletion mutants of *mexZ*, *armZ*, and *parRS* in the PAO1-Bes background (Table 2). Deletion of *mexZ* or *armZ* did not alter susceptibility to NOSO-502, whereas inactivation of *parRS* resulted in a modest, 2-fold MIC decrease. In contrast, NOSO-95C and gentamicin displayed altered activity profiles in the mutants. Deletion of *mexZ* led to 4- to 8-fold MIC increases for both NOSO-95C and gentamicin, while deletion of *armZ* caused a 4-fold reduction in MICs. The MICs of NOSO-95C and gentamicin were unaffected by *parRS* deletion. These findings suggest that NOSO-95C, but not NOSO-502, may induce *mexXY* expression *via* a pathway dependent on MexZ and ArmZ.

**Fig 2:**
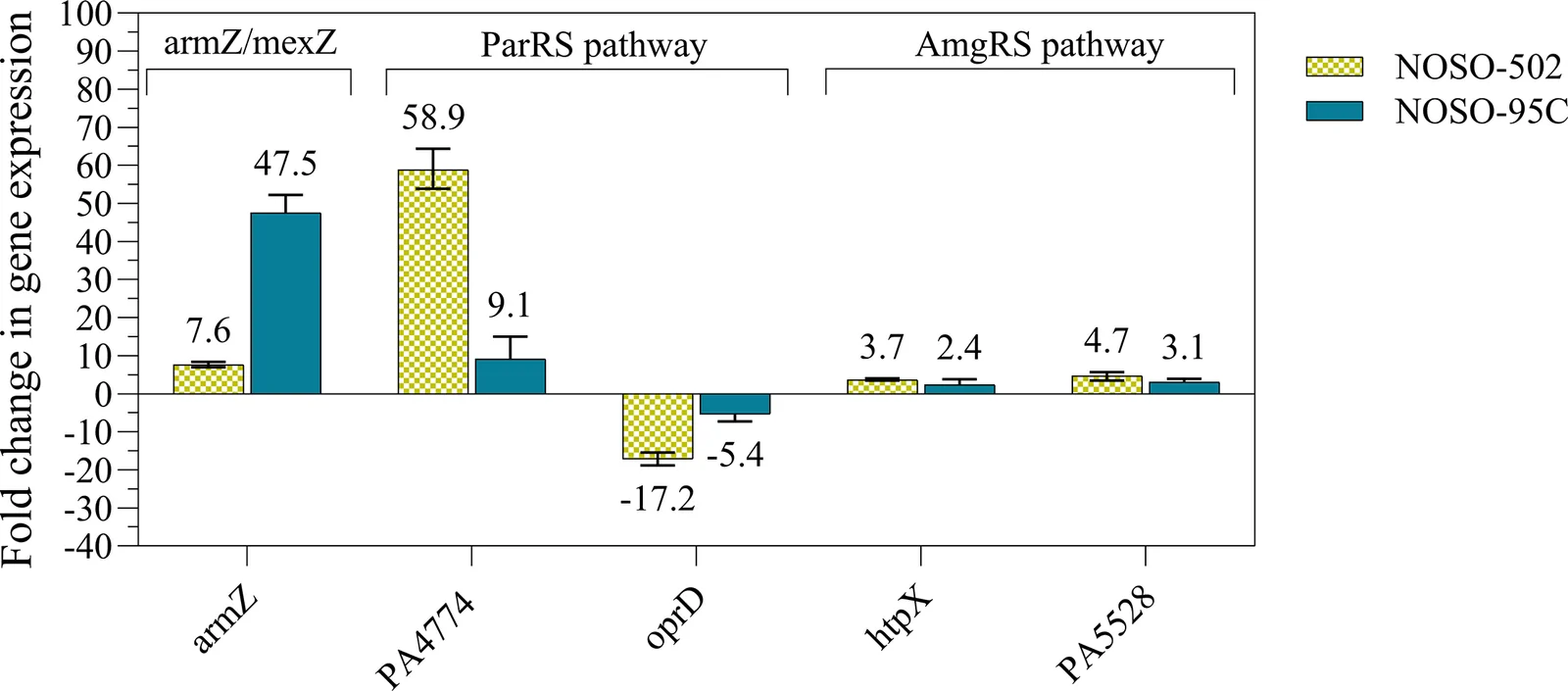
RT-qPCR comparative analysis of armZ (ArmZ/MexZ pathway), PA4774 and oprD (ParRS pathway), and htpX and PA5528 (AmgRS pathway) gene expression in PAO1 cultures treated with NOSO-502 or NOSO-95C (128 µg/mL, 45 min) relative to untreated cultures. Mean values from three biological replicates are shown, and error bars represent the SEM.

We then focused on the expression of genes involved in known mexXY induction pathways, using RT-qPCR to supplement our initial findings (30, 31). Interestingly, while NOSO-502 and NOSO-95C induced the AmgRS markers htpX and PA5528 at similar levels (3.1-fold to 4.7-fold), their effects on other pathways diverged significantly. For instance, NOSO-502 triggered a major response in ParRS markers, notably a 17.2-fold repression of oprD and a 58.9-fold induction of PA4774, whereas armZ (the MexZ anti-repressor) showed only moderate induction (7.6-fold). Conversely, NOSO-95C-treated cells showed a strong 47.5-fold induction of armZ; yet the impact on ParRS markers remained comparatively weak (5.4-fold repression of oprD and 9.1-fold induction of PA4774) (Fig. 2).

### Periplasmic depletion of the inactive NOSO-502

**Fig 3:**
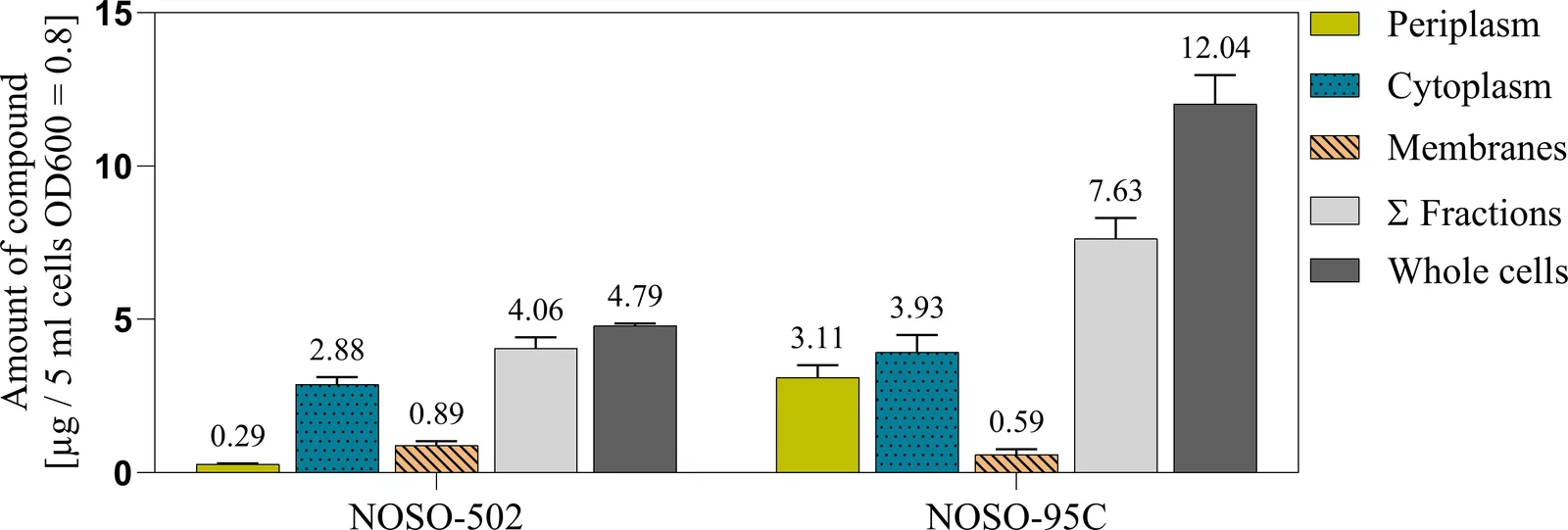
Comparison of NOSO-502 and NOSO-95C uptake in PAO1 at 128 µg/mL for 45 min. The periplasm fraction is shown in green, the cytoplasm in blue, the membranes in pink striped, the sum of the fractions in light gray, and the whole-cell sample in dark gray. Average values of at least two biological replicates with two technical replicates each are depicted, and the error bars show the SEM.

To evaluate the consequence of the differential *mexXY* induction by NOSO-502 and NOSO-95C on their uptake in *P. aeruginosa* PAO1, the amount of both compounds in the various compartments of the cell was determined by applying a bioanalytical method that combines cellular fractionation with quantitative mass spectrometry (35). Absolute amounts of NOSO-502 (tested at 128 µg/mL) internalized after 45 min of incubation were measured and confirmed its uptake in different subcellular compartments of *P. aeruginosa* PAO1. The largest amount of NOSO-502 (2.88 µg [± 0.02 µg]) was recovered in the cytoplasm, while lower amounts were present in the periplasm (0.29 µg [± 0.23 µg]) and the membrane fractions (0.89 µg [± 0.14 µg]) (Fig. 3). Using the same conditions, the concentration of NOSO-95C in cytoplasm was found to be similar to the one evaluated for NOSO-502 (3.93 µg [± 0.57 µg]) as well as the amount in membranes (0.59 µg [± 0.18 µg]), while the amount found in periplasm was 10.7-fold higher (3.11 µg [± 0.40 µg]) (Fig. 3).

### Structure-activity relationships between ODL analogs and MexXY-OprM-mediated resistance in *P. aeruginosa*

To understand the structural determinants of ODLs susceptibility to the MexXY-OprM efflux system, a medicinal chemistry program was initiated. We focused on two parameters: the ability of NOSO-502 and NOSO-95C to induce *mexXY* expression (measured by RT-qPCR after 45 min exposure of *P. aeruginosa* PAO1), and their susceptibility to efflux, expressed as a MIC ratio (MIC in PAO1/MIC in the efflux-deficient PAO1-11B strain).

**Fig 4:**
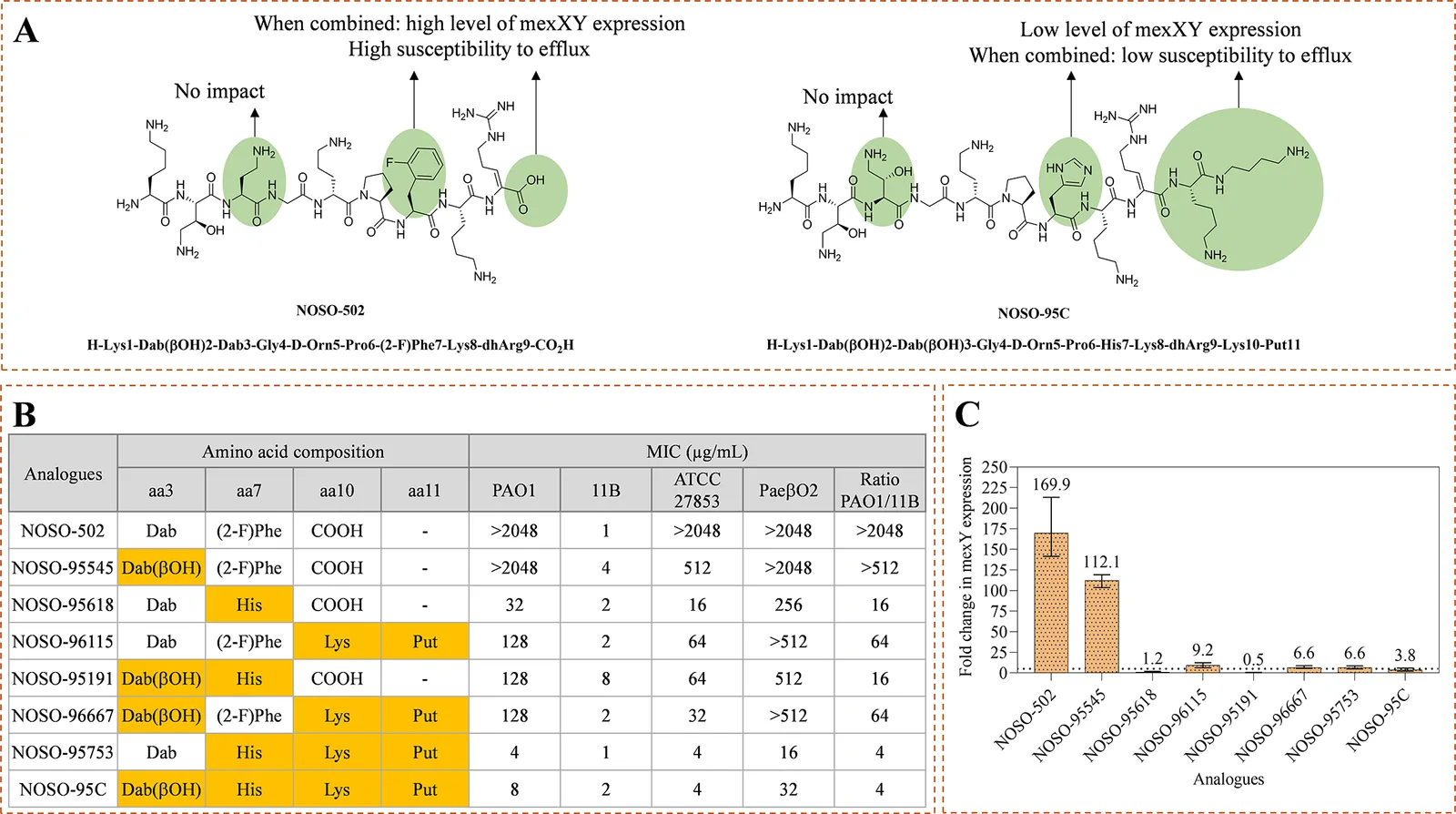
Medicinal chemistry strategy to understand antibacterial activity of odilorhabdins against *P. aeruginosa*. (**A**) Structures of NOSO-502 and NOSO-95C and summary of structure-activity relationship (SAR) findings. (**B**) Relationships between ODL structures and antibacterial activity against *P. aeruginosa* wild-type strain PAO1 and 11B (PAO1 MexXY-OprM-deficient mutant). Ratio PAO1/11B: MIC value against PAO1 related to MIC value against 11B indicates if the activity remains impacted by the MexXY-OprM efflux pump. aa, amino acid; Put, Putrescine (1,4-diaminobutane). (**C**) RT-qPCR comparative analysis of the *mexY* gene differentially expressed in PAO1 cultures treated with NOSO-502, NOSO-95C, or analogs (128 µg/mL, 45 min) relative to the culture without treatment. Average values of three biological replicates are depicted, and the error bars show the SEM.

NOSO-95C is a 10-mer linear cationic peptide composed of six proteinogenic and four non-standard amino acids (Fig. 4A) (10). NOSO-502 differs by three modifications: Dab(βOH)3 is replaced by Dab, His7 by 2-fluoro-phenylalanine ((2-F)Phe), and the *C*-terminal Lys10 and Put11 residues are deleted, leading to a *C*-terminal carboxylic acid function (Fig. 4A).

Each of these three changes was first evaluated independently. All resulting analogs retained intrinsic antibacterial activity against strain 11B (MICs ranging from 1 to 8 µg/mL) (Fig. 4B). Replacement of Dab3 by Dab(βOH)3 (analog NOSO-95545) had minimal effect on *mexXY* expression (<2-fold difference) and efflux susceptibility (Fig. 4B and C). Substitution of (2-F)Phe7 by His7 (NOSO-95618) drastically reduced *mexXY* expression (>100-fold) and markedly improved the MIC ratio (>128-fold). However, the MIC ratio remained high, indicating significant residual efflux activity.

Reintroducing Lys10-Put11 (NOSO-96115) also led to a strong reduction in *mexXY* expression (>10-fold) and MIC ratio (>32-fold), although the ratio remained substantial.

Double modifications were then assessed. The combined changes did not further reduce *mexXY* expression significantly but did affect efflux susceptibility. The addition of Dab(βOH)3 had little impact (e.g*.,* NOSO-95191 vs. NOSO-95618), whereas combining His7 and Lys10-Put11 (NOSO-95753) resulted in a synergistic effect, reducing the MIC ratio to 4, similar to what was observed with NOSO-95C.

These findings show that specific residues at positions 7 and 10–11 modulate both the ability of ODLs to induce *mexXY* expression and their recognition as efflux substrates, which, in turn, shape their antibacterial potency against *P. aeruginosa*.

## DISCUSSION

This study provides new insights into the intrinsic resistance mechanisms of *P. aeruginosa* to ODLs, a novel class of ribosome-targeting antibiotics. Our comparative evaluation of NOSO-502 and NOSO-95C highlights the complex interplay between compound structure, efflux susceptibility, and gene expression modulation in determining ODLs’ efficacy against this clinically relevant gram-negative pathogen.

Our data demonstrate that *P. aeruginosa* is highly resistant to NOSO-502, with MIC values ≥ 512 µg/mL in the PAO1 strain and ≥64 µg/mL across all isolates tested. This resistance was clearly linked to the MexXY-OprM efflux system, as the deletion of *mexXY* or *oprM* resulted in >256-fold MIC reductions. This aligns with the known substrate profile of MexXY-OprM, which preferentially extrudes ribosome-targeting antibiotics such as aminoglycosides.

Interestingly, NOSO-95C showed variable activity across the isolate panel, with intermediate susceptibility observed in PAO1 (MIC = 4–8 µg/mL). While this compound also remained a substrate of MexXY-OprM, its lower capacity to induce *mexXY* expression appears to mitigate efflux-mediated resistance. RT-qPCR analyses revealed a 170-fold induction of *mexY* by NOSO-502 compared to a modest 3.8-fold by NOSO-95C at equivalent concentrations, suggesting a direct relationship between induction potential and resistance phenotype.

The differential expression of *mexXY* by the two ODLs is a key finding. By triggering strong *mexXY* overexpression, NOSO-502 induces its own efflux, creating a feedback loop that exacerbates its inactivity in *P. aeruginosa*. This phenomenon was further confirmed by co-treatment assays: NOSO-502 enhanced resistance not only to itself but also to gentamicin and NOSO-95C, supporting the hypothesis of efflux induction as a resistance amplifier. The use of berberine, a MexXY inhibitor, validated this mechanism and partially restored the activity of both compounds. The modest increases in MICs observed in the presence of PAβN (2-fold to 4-fold for ODLs and 2-fold for gentamicin) may be explained by its ability to transiently depolarize the inner membrane at the concentrations used in this study (36), inducing a limitation of the proton-motive force required for the uptake of both antibiotics (37).

Our results suggest that NOSO-95C induces mexXY through the ArmZ-MexZ pathway. Indeed, the deletion of armZ (MexZ anti-repressor) led to a 4-fold increase in susceptibility (MIC decreased from 4 to 1 µg/mL), while the loss of mexZ (mexXY repressor) resulted in an 8-fold increase in MIC values (from 4 to 32 µg/mL). These shifts are consistent with the potent induction of armZ (47.5-fold) observed by RT-qPCR. On the other hand, NOSO-502 resistance appears less dependent on this pathway. Indeed, MICs remained high and seem unaffected in armZ and mexZ mutants (MIC ≥512 µg/mL), with a moderate induction of armZ expression compared to NOSO-95C (7.6-fold). However, the ParRS system seems partially involved in the response to NOSO-502. Its deletion led to a > 2-fold decrease in MIC (with no effect on NOSO-95C) and was associated with a pronounced ParRS transcriptional signature (*PA4774*: 58.9-fold; *oprD*: −17.2-fold). Both compounds induced the expression of the AmgRS-dependent markers htpX and PA5528 to a similar extent (3.1-fold to 4.7-fold). This comparable activation suggests that the AmgRS pathway is unlikely to be involved in the distinct susceptibility profiles observed between NOSO-502 and NOSO-95C. Nevertheless, the direct role of this system was not assessed using an amgRS mutant, which represents a limitation of the present study. The high level of resistance of *P. aeruginosa* PAO1 to NOSO-502 is clearly linked to MexXY-OprM, but the regulatory pathways governing the expression of this pump in response to this compound remain to be fully characterized.

The compartmental distribution of the drugs within *P. aeruginosa* cells offers additional mechanistic insight. Although both NOSO-502 and NOSO-95C were detected in the cytoplasm and membranes, the periplasmic accumulation of NOSO-502 was substantially lower, 10.7-fold less than that of NOSO-95C after 45 min of exposure. This periplasmic depletion likely reflects the combined effect of MexXY-OprM overexpression induced by NOSO-502 and its susceptibility to efflux, consistent with the mode of action of certain efflux pumps (30). Interestingly, the cytoplasmic levels of NOSO-502 and NOSO-95C were comparable at this early time point (NOSO-502 being only 1.4-fold lower), suggesting that efflux cannot completely prevent initial translocation into the cytoplasm. However, this snapshot likely underestimates the cumulative impact of efflux over the 24-h duration of standard MIC assays. We hypothesize that over longer exposure periods, sustained efflux activity, driven by persistent *mexXY* overexpression, leads to progressive depletion of NOSO-502 in the cytoplasm, ultimately compromising antibacterial efficacy.

To further delineate the molecular determinants of ODL susceptibility, we employed a structure-activity relationship (SAR) approach. Our medicinal chemistry program identified two critical regions influencing *mexXY* induction and efflux susceptibility: the residue at position 7 and the *C*-terminal extension. Substitution of (2-F)Phe7 with His7 drastically reduced *mexXY* induction, while restoration of the Lys10-Put11 tail, absent in NOSO-502, diminished susceptibility to efflux. Importantly, combining both modifications synergistically enhanced activity in wild-type PAO1 and reduced efflux dependence, as illustrated by the low MIC ratio of NOSO-95753 and NOSO-95C.

Taken together, our findings suggest that structural features of ODLs directly impact their ability to induce *mexXY* expression and determine their vulnerability to efflux. This dual effect, on gene regulation and substrate recognition, poses a significant challenge for the design of ODLs active against *P. aeruginosa*. Nonetheless, the partial activity of NOSO-95C and its analogs provides a promising starting point for the rational development of new ODL derivatives with optimized efflux evasion properties.

In conclusion, our study underscores the importance of efflux pump induction as a resistance mechanism and highlights the potential of structural tuning to overcome it. Future ODL optimization efforts should prioritize minimizing *mexXY* induction and reducing affinity for the MexXY-OprM system, paving the way toward effective treatments for infections caused by multidrug-resistant *P. aeruginosa*.

## MATERIALS AND METHODS

### Bacterial strains used in this study

*P. aeruginosa* reference strains were purchased from the American Type Culture Collection (ATCC), the National Collection of Type Cultures (NCTC), and the Institut Pasteur Collection (CIP). *P. aeruginosa* ATCC 27853 (ST-155) was collected from human blood culture (38); *P. aeruginosa* CIP 106816 was collected from human urine and produces an adenylyltransferase AAD-A6 and an extended-spectrum beta-lactamase VEB-1 (39); *P. aeruginosa* CIP 106817 was collected from human urine and is resistant to imipenem and all aminoglycosides except gentamicin by producing a carbapenemase VIM-2 and an aminoglycoside acetyltransferase AAC 29 (40); *P. aeruginosa* CIP 106880 was collected from human bile and produces beta-lactamases OXA-18 and OXA-20 (41); *P. aeruginosa* NCTC 13437 is an MDR strain, resistant to carbapenems and other β-lactam antibiotics, harboring the VEB-1 extended-spectrum β-lactamase (ESBL) and the VIM-10 MBL (42). Pseudomonas strains 1003 682 729, 1004 049 478, 1004 091 973, 1004 131 924, 1004 141 296, 1004 75 25, 2004 149 316, 35170, 401681, and 5618 were collected from patients of Nîmes University Hospital.

*P. aeruginosa* strains PA01, PAO1-Bes, and PAO1-derived mutants used in this study are described in Table 2.

### Antimicrobial agents

NOSO-502 and analogs were synthesized at Nosopharm (Nîmes, France). Ciprofloxacin (AC58172, Biosynth), gentamicin (G1397-10ML, Sigma-Aldrich), imipenem (IO160, Sigma-Aldrich), cefepime hydrochloride (A3737, Sigma-Aldrich), and tigecycline (PZ0021, Sigma-Aldrich) were provided as standard powders by the manufacturers.

### Minimum inhibitory concentration

MIC values were determined using the Clinical and Laboratory Standards Institute (CLSI) broth microdilution (BMD) methodology, colony direct suspension, as described in CLSI document M07-A10 (43).

### Measurement of uptake of odilorhabdins into *P. aeruginosa* by LC-MS

#### Cell fractionation

The strain *P. aeruginosa* PAO1 was pre-cultured in cation-adjusted Müller-Hinton medium (Merck-Millipore, Darmstadt, Germany) overnight at 37°C, shaking at 150 rpm. A new culture was set up the following day by diluting the pre-culture to an OD_600_ = 0.2 in medium and grown until OD_600_ = 0.8 (logarithmic growth phase). The procedure for cell fractionation into periplasm (PP), cytoplasm (CP), and membranes (M) was modified from a study by Prochnow et al. (35). Briefly, 10 mL culture at OD_600_ = 0.8 was portioned into 15 mL conical flasks and treated with the test compound at 128 µg/mL concentration dissolved in water. The culture was incubated with the compound for 45 min at 37°C, 150 rpm (shaking). Next, the cells were harvested by centrifugation at 4,500 × *g* for 5 min at 4°C and resuspended in 2 mL TBS (50 mM Tris-HCl, pH 7.0, 135 mM NaCl, 2.5 mM KCl). This suspension was divided equally into two 2 mL Eppendorf tubes. The first part of the culture was used to generate the whole-cell sample, and the other part was used for fractionation. Modifications to the published protocol were that buffers containing a higher salt concentration were used for washing and sucrose treatment, as this provided for easier handling of *Pseudomonas* samples compared to the previously reported method. In detail, for washing, TBS was used instead of 25 mM Tris pH 7.4, and for the TS washing step after incubation with the sucrose-EDTA, TBS with 20% sucrose (wt/vol) was used instead of Tris buffer containing sucrose. Samples mock-treated with water instead of the compound solutions were used to generate matrix standard curves. Typically, each compound was used to treat at least two different biological replicates, which were processed in two technical replicates each. All samples were stored at −20°C until preparation for LC-MS/MS analysis.

#### LC-MS/MS analysis

The preparation of the samples for LC-MS/MS analysis was optimized specifically for odilorhabdins. Either 80 µL of the sample or a dilution of a standard in mock-treated matrix was pipetted into a 96-well deep well plate (Brand, BR701354), 10 µL of 50% (wt/vol) trichloroacetic acid (TCA) and 10 µL of the 10× internal standard solution, providing for final concentrations of 10% (vol/vol) acetonitrile (ACN), 0.4% (vol/vol) heptafluorobutyric acid (HFBA), and 10 ng/mL caffeine were added. The samples were mixed briefly and then centrifuged for 60 min at 2,250 × *g* to separate precipitated proteins from the sample; 80 µL of the clarified supernatant was transferred to a MTP plate with V-shaped wells (Greiner Bio-One, 651201) and measured on a triple quadrupole mass spectrometer (QTrap 6500, AB Sciex Germany GmbH, Darmstadt, Germany) in positive mode. To this end, 5 µL of each sample were separated using a Symmetry C18 column, 300Å, 3.5 µm, 2.1 mm × 50 mm equipped with a Symmetry C18 Sentry Guard Cartridge, 300 Å, 3.5 µm, 2.1 mm × 10 mm (Waters, Germany) using a gradient of water with 0.1% (vol/vol) formic acid, 0.1% (vol/vol) acetic acid, 0.04% (vol/vol) HFBA, and acetonitrile with 0.05% (vol/vol) formic acid. In brief, the LC profile consisted of a 1 min step at 95% H_2_O, 5% ACN, followed by a gradient from 5% to 95% ACN over 4 min with a final step for 1 min at 95% ACN using a flow rate of 0.8 mL/min while heating the column to 50°C. Details of the transitions used to detect the compounds are given in Table S1. Data analysis was performed using MacCoss Lab Software Skyline Targeted Mass Spec Environment from the University of Washington (2023).

### Determination of mRNA expression levels by RT-qPCR

Overnight culture of strain PAO1 in Mueller Hinton Broth (Becton Dickinson, ref: 212322) was diluted 2:100 into a fresh medium and cultivated at 37°C until OD_600nm_ of 0.5–0.6; 128 µg/mL of NOSO-502, NOSO-95C, or analogs were added to PAO1 cultures before incubation with vigorous shaking at 37°C for 45 min (induction phase). Free-drug culture of PAO1 (used as control) was also incubated at 37°C for 45 min with vigorous shaking. Total mRNA extraction was achieved with the RNeasy Protect Bacteria 50 preps kit (Qiagen ref. 74524) according to the manufacturer’s instructions and was performed on three independent biological replicates. RNA Integrity Number was determined, and reverse transcription was performed using SuperScript II Reverse Transcriptase (Invitrogen ref. 18064-022) and random hexamer from Applied Biosystems ref. N8080127. RT-qPCR to follow mexY, oprM, and the expression of other genes was carried out using a LightCycler 480 (Roche) with Sensi-Fast SYBR no rox commercialized by Bioline (BIO-98050) and with primers MexY1a (5′-CTA CAA CAT CCC CTA TGA CAC CTC-3′) and MexY1b (5′-ATGGTCAGCACGTTGATCGAGAA-3′), OprM1a (5′-GCCTGGGAACTCGATCTCTTC-3′) and OprM1b (5′-GTCAGGTCGAAACTCTTCTGGTAG-3′), OprD1a (5′-CATCTACCGCACAAACGATG-3′) and OprD1b (5′-CAGAGTTGGCGAGGAAAATC-3′), PA47741a (5′-CTCTCTATACCCGCCAGTTCTAC-3′) and PA47741b (5′-TAGCTGTGGACCTGGGAGAAG-3′), ArmZ1a (5′-TCGAGGTAATCGAGGAGGTG-3′) and ArmZ1b (5′-AGGGTCTGCAAACGGATCTC-3′), htpX1a (5′-CTTCACCGGCCAGAATTACG−3′), and htpX1b (5′-GACAGCTCTTCGACGGTTTG−3′), *PA5528*1a (5′-GGGATCAGTTCTGCCGGATA−3′) and *PA5528*1b (5′-CTACCAGTCCACCTACATCGAC−3′). Gene expression was normalized to that of untreated strain PAO1 after internal normalization with gene *rpsL*, taken as a reference housekeeping gene (using REST software 2009 and the Pfaffl equation (44). The data presented are means of at least three independent experiments. As controls, a blank sample (distilled water) and a no reverse transcriptase control were included to exclude DNA contamination.

## Data Availability

All data generated or analyzed during this study are included in this article and its supplemental material.

## SUPPLEMENTAL MATERIAL

Table S1 spectrum.00466-26-s0001.docx Mass spectrometric conditions used for MS/MS detection of odilorhabdins.

ASM does not own the copyrights to Supplemental Material that may be linked to, or accessed through, an article. The authors have granted ASM a non-exclusive, world-wide license to publish the Supplemental Material files. Please contact the corresponding author directly for reuse.
