# Supplementary material for: MexXY-OprM efflux pump mediates resistance to odilorhabdins in Pseudomonas aeruginosa
Source: Microbiol Spectr. 2026 Jun 16;14(7):e00466-26. doi: 10.1128/spectrum.00466-26 (PMC13340244; doi:10.1128/spectrum.00466-26)
Supplement: Table S1 — Mass spectrometric conditions used for MS/MS detection of odilorhabdins. [file spectrum.00466-26-s0001.docx]

| Compound | Q1 mass  [g/mol] | Q3 mass  [g/mol] | Time  [ms] | Declustering  potential [V] | Entry potential [V] | Collision energy [V] | Collision cell exit potential [V] |
| --- | --- | --- | --- | --- | --- | --- | --- |
| **Cafeine** |  |  |  |  |  |  |  |
| Quantifier | 195.1 | 138.1 | 40 | 66 | 10 | 27 | 10 |
| Qualifier | 195.1 | 110.1 | 40 | 66 | 10 | 31 | 6 |
| **NOSO-502** |  |  |  |  |  |  |  |
| Quantifier | 360.3 | 282.2 | 40 | 1 | 10 | 29 | 40 |
| Qualifier | 360.3 | 70.1 | 40 | 1 | 10 | 29 | 42 |
| **NOSO-95C** |  |  |  |  |  |  |  |
| Quantifier | 422.4 | 416.3 | 40 | 1 | 10 | 21 | 30 |
| Qualifier | 422.4 | 115.2 | 40 | 1 | 10 | 31 | 4 |

**Supplementary Table S1**: Mass spectrometric conditions used for MS/MS detection of odilorhabdins.
